# Caregiver interactions, perceived control, and meaning in life of elderly: the moderating effect of the elderly-to-social worker ratio

**DOI:** 10.1186/s12877-024-05029-7

**Published:** 2024-05-15

**Authors:** Xiaofan Zhou, Hung Wong

**Affiliations:** 1 School of Sociology, Central China Normal University, Wuhan, China; 2 Department of Social Work, The Chinese University of Hong Kong, Shatin, The New Territories, Hong Kong, China

**Keywords:** Institutional care, Caregiver interaction, Perceived control, Meaning in life, Social worker

## Abstract

**Background:**

Meaning in life is a widely accepted aim in promoting psychosocial health in institutional care. However, how caregiver interaction and perceived control impact meaning in life among the elderly remains unclear. This study explores the effect of institutional caregiver interaction, family caregiver interaction, and perceived control on meaning in life among elderly residents in China, and the potential moderating effect of elderly-to-social worker ratio in these associations.

**Methods:**

Multistage random sampling was used to recruit a sample of 452 elderly residents from 4 elderly care homes in urban China. A structural equation model was used to test the study hypothesis.

**Results:**

Institutional caregiver interaction is positively related to meaning in life, and perceived control among elderly residents has a positive impact on meaning in life. Moreover, the elderly-to-social worker ratio moderated the relationship between institutional caregiver interaction and meaning in life, as well as between family caregiver interaction and meaning in life.

**Conclusions:**

Increase elderly’s meaning in life is an important service target for the caring professions in institutional care. Social workers affect the effectiveness of interventions on elderly’s meaning in life in institutional care. A higher elderly-to-social worker ratio could improve the effectiveness of interventions on meaning in life for elderly residents.

## Introduction

The rapid development of population aging brings social and life changes, which exacerbate the mental health needs of older adults in institutional care [1]. Finding meaning in life is one of the most important factors in promoting mental health [2]. Compared with older adults who are cared for in the community, residents of elderly care institutions are more vulnerable in the perception of meaning in life [3]. Retaining resident’s meaning in life could help them deal with suffering and distress, cope with loss, and reduce the risk of suicide [4, 5]. However, few studies have discussed meaning in life in institutional settings, with most studies involving elderly people living in communities [6]. Consequently, perceptions of meaning in life among elderly residents in institutional care are the emerging focus of this study.

According to the ethic of care theory, caregiver interaction and perceived control is an important strategy for elderly adults to retain meaning in life [7]. Many studies have shown that perceived control can reduce levels of anxiety and depression [8]. Some discussions have examined the relationship between institutional caregiver interactions and meaning in life [9, 10], without considering the impact from family caregivers at the same time. What’s more, the relationship between family caregiver interaction and meaning in life remains unclear. Some scholars believe that family does not significantly affect actual perceptions of meaning in life in later life stage [11]. Other studies suggest that the quality of family relationships contributing to greater meaning in life [12]. Understanding the impact from family caregiver interaction, institutional caregiver interaction and perceived control could provide an effective intervention guideline for enhancing resident’s meaning in life.

Tronto extended ethic of care theory at the organizational level, by stating that human services professionals—such as social workers—could promote the humanistic environment to improve the service quality [13, 14]. Because residents are struggling to find meaning in life in care facilities, they need adequate social workers to build a supporting system for the provision of humanistic care [15]. The importance of social work service has been recognized by the policy maker in China. Several major cities such as Beijing, Nanjing, Chongqing and Wuhan have implemented standard of 1% elderly-to-social worker ratio for evaluating the quality of elderly homes. Prior studies on meaning in life have mainly focused on the direct social work intervention effect [16, 17], but little research has been undertaken to determine how the 1% elderly-to-social worker ratio could impact the provision of intervention in Asian culture. For this reason, the present study aims to investigate the association between caregiver interactions, perceived control, and meaning in life in institutional care settings by considering the elderly-to-social worker ratio as a moderating factor.

## Theoretical framework and hypotheses

### Factors predicting meaning in life

#### Institutional caregiver interactions and meaning in life

Institutional caregiver interactions represent a moral practice of person-centered values between service providers and users in elderly care homes. They require that institutional staff pay attention to understand, support, and recognize elderly care recipients, which helps them gain a sense of trust, security, identity, and happiness [18]. Elderly interaction with institutional staff includes verbal and behavioral interactions, both of which can effectively improve perceptions of meaning in life among the elderly [19, 20]. Based on the existing literature, which suggests that institutional caregiver interactions have a positive relationship with meaning in life among the elderly, this study proposes the following hypothesis:

##### Hypothesis 1 (H1)

Positive institutional caregiver interactions have a positive impact on meaning in life among the elderly.

#### Family caregiver interactions and meaning in life

Family caregiver interactions refer to the frequency of communication between elderly care residents and their family members after they relocate to the elderly care home. The origins of meaning in life concept highlighting the importance of family member interactions [21]. Within an Asian cultural environment that values filial piety, the emotional support that the elderly receive through interactions with family caregivers can strengthen their sense of meaning in life [22]. The above evidence suggests that family caregiver interactions may have a positive relationship with meaning in life among the elderly. Therefore, this study proposes the following hypothesis:

##### Hypothesis 2 (H2)

Positive family caregiver interactions have a positive impact on meaning in life among the elderly.

#### Perceived control and meaning in life

Referring to the belief of having a clear sense of structure and order, sense of control predicts a stronger perception of meaning in life [23]. The ethics of care theory encourages caregivers to fulfill the social and emotional needs of elderly care residents [24], thus allowing them to retain a certain sense of control over their environment and daily activities, which in turn improves their spiritual wellbeing [25]. Additionally, many researchers have found a positive relationship between control and meaning in life among adolescents [26, 27]. Based on the ethics of care theory and research evidence, this study proposes the following hypothesis:

##### Hypothesis 3 (H3)

Positive perceived control has a positive impact on meaning in life among the elderly.

### Moderating role of elderly-to-social worker ratio

Social workers facilitate communication, autonomy and safe team culture [28], which could facilitate service quality indirectly [29]. The Higher social work staff ratio resulted in greater effectiveness for institutional caregiver interventions on elderly mental health [30]. Lower elderly-to-social worker ratio will lead to heavy workload and insufficient responses, which may impact the relationship between institutional caregiver interaction and perceived control to meaning in life [31]. Social workers play a role that supports the impact between family caregiver interactions and meaning in life. Based on the Chinese government’s guideline for suitable elderly-to-social worker ratio, this study uses 1% as the dividing line to compare the moderating effect between higher elderly-to-social worker ratio group and lower elderly-to-social worker ratio group. The following three hypotheses are proposed:

#### Hypothesis 4 (H4)

A higher elderly-to-social worker ratio will strengthen the positive association between institutional caregiver interactions and elderly residents’ sense of meaning in life.

#### Hypothesis 5 (H5)

A higher elderly-to-social worker ratio will strengthen the positive association between family caregiver interactions and elderly residents’ sense of meaning in life.

#### Hypothesis 6 (H6)

A higher elderly-to-social worker ratio will strengthen the positive association between elderly residents’ perceived control and their sense of meaning in life.

## Methods

Developed from the early experiences of attachment, the ethics of care theory emphasizes that institutional caregiver interaction, family caregiver interaction, and perceived control can help elderly adults adapt to the aging process, which is an important strategy for retaining meaning in life [32]. The elderly-to-social worker ratio could impact caregivers’ understanding of the vulnerable group’s needs, thereby strengthening or weakening the interventions on meaning in life [33]. This study aims to explore the direct effect from the individual level and the moderating effect from the organizational level on meaning in life in institutional care. By measuring with scales and conducting data analysis, it is possible to better analyze the differences in the weighting of different factors’ impacts.

### Sample and data collection procedures

Guided by the research hypotheses, this study focuses on the elderly who receive social work services at elderly care homes in urban areas. Multistage random sampling method was used to collect data in a central urban city in China from August to December 2019.

In stage one, all 198 registered elderly care homes in the city were set as the study population. of which 193 institutions were excluded as they did not hire full-time registered social workers to provide social services such as casework, group work and activity for residents.

In stage two, the 5 long-term care institutions with registered social workers are divided into two groups according to the elderly-to-social worker ratio. The number of registered social workers represents the staff who provide social work services with professional certificate. And the data of elderly residents is collected based on the name list provided by the elderly homes. 2 elderly homes were randomly selected among the institutions where the elderly-to-social worker ratio is higher than 1% (for one, 4 social workers to 338 residents; the other, 4 social workers to 283 residents), and 2 elderly homes were randomly selected among the institutions where the elderly-to-social worker ratio is lower than 1% (for one, 3 social workers to 313 residents; the other, 3 social workers to 490 residents).

In stage three, 120 elderly residents from each elderly home were randomly selected from the resident lists. The inclusion criteria for selection were as follows (1) residents age 60 or above; (2) residents who had lived at the current care institution for at least 6 months; and (3) residents who had not been diagnosed with dementia or other mental illnesses. Experienced interviewers collected data through face-to-face interviews. Among the 480 elderly residents recruited for the study, 28 declined to participate or could not be contacted, which resulted in a final sample of 452 elderly residents with a 94.2% response rate. Since no case had missing values, the questionnaire responses for all 452 respondents were included in the final analysis.

The social-demographic variables that might have potential impact on meaning in life were chosen as control variables, such as age [34], gender [35], duration of stay [10], education level [36], marital status [21] and income level [37]. Among the sample, 35% of the elderly residents were male and 65% were female. In terms of age, 37.8% of elderly residents were aged between 86 and 90, 33.2% were aged between 81 and 85, 19.5% were aged 80 or below, and 9.5% were aged 91 or above. Table 1 presents descriptive statistics for the social-demographic variables of the sample.

**Table 1:** Social-demographic information of participants (*N* = 452)

	Frequency	Percent
Gender		
Male	158	35
Female	294	65
Age		
	M = 84.41	SD = 5.76
Duration of stay		
	M = 43.33	SD = 41.73
Education level		
Illiteracy	37	14.5
Literacy	84	18.6
Primary school	52	11.5
Junior high school	82	18.1
Senior high school	99	21.9
Bachelor or higher	98	21.6
Marital status		
Married or single	110	24.3
Widowed	342	75.7
Income level		
Difficult	72	15.9
Make ends meet	170	37.6
Have saving	210	46.5

### Measurement instruments

#### Institutional caregiver interaction

Guided by the person-centered care framework, the Nurse-Patient Interaction Scale (NPIS) shortened version specifically for quantifying the characteristics of caregiver interactions in elderly homes [38]. The term “nurse” encompasses all employees providing care services at nursing homes, including nurses, care workers, and social workers. NPIS comprises eight questions scored using a 10-point scale from 1 (completely disagree) to 10 (completely agree). Elderly home residents gave scores according to their attitudes about their relationships with care service providers, with a higher score indicating a better relationship between residents and their caregivers. In the present sample, the NPIS showed high internal consistency based on the value for Cronbach’s alpha (α = 0.956).

#### Family caregiver interaction

Family caregiver interaction was measured using the Frequency of Interaction with Family Scale (FIFS), a three-question scale that evaluates three types of interactions with family caregivers: family visits to care homes, telephone communications, and elderly visits to family homes [39]. Living in the elderly home affects the relationship between the elderly and their families in terms of the level of interaction with their family caregiver, which may remain stable, improve, or deteriorate. Each question item is scored on a 3-point scale (1 = never, 2 = sometimes, 3 = every day), with a higher score referring to a higher level of interaction. In the present sample, the FIFS showed acceptable internal consistency based on the value for Cronbach’s alpha (α = 0.551).

#### Perceived control

The environment in which an individual lives greatly influences their sense of control. The Perceived Control Scale (PCS) was used to measure the sense of control in an institutional care environment, interviewers asked residents three questions regarding the extent of control they perceive that they have in their daily activities, daily living arrangements, and social life [24]. Interviewees gave scores on an 11-point scale from 0 (completely disagree) to 10 (completely agree), with a higher score indicating higher levels of perceived control. In the present sample, the question set showed high internal consistency based on the value for Cronbach’s alpha (α = 0.945).

#### Meaning in life

Meaning in life refers to the ability to transcend acceptance and attitudes towards life at the individual level and participate in physical, psychological, and social activities [4]. Meaning in life-oriented care services encourage professionals to focus on the psychological dimensions of older people and respond more positively to the challenges of aging. Since the content contributing to meaning in life may differ across various cultures, it is better to understand these perceptions according to the personal contexts of elderly residents [21]. This study used the Meaning in Life Questionnaire (MLQ), which was designed specifically for the elderly in Chinese society [40], to assess perceptions of meaning in life among surveyed residents. The MLQ comprises five items that use a 5-point Likert scale (1 = never, 5 = always), and includes questions about existence and the pursuit of meaning in life. A higher score represents a stronger sense of meaning in life. In the present sample, the MLQ showed good internal consistency based on the value for Cronbach’s alpha (α = 0.898).

#### Elderly-to-social worker ratio

This study operationalizes the social worker ratio as the elderly-to-social worker ratio. The number of social workers was determined based on the actual number of registered social worker at each care home, while the number of elderly residents refers to the actual number of residents living at each care home during the study period. Since the sample was collected in Hubei province, this study will take the recommended standard from the local government of elderly-to-social worker ratio in 1% as the dividing line. Therefore, the sample was divided into two groups based on the ratio for each care home. The high-ratio group includes two elderly homes with elderly-to-social worker ratios of 1.183% and 1.413%, respectively, while the low-ratio group includes the other two elderly homes with elderly-to-social worker ratios of 0.958% and 0.612%, respectively.

### Data analysis

Questionnaire and interview data were processed using structural equation modeling (SEM) in SPSS Amos 24, while descriptive statistics summarizing the demographic information of the elderly sample were generated in SPSS 25. The data analysis process included the social-demographic variables of gender, age, duration of stay, education level, marital status, and income level as control variables.

The analysis of the direct effect model includes two stages. First, confirmatory factor analysis (CFA) was performed to measure the construct validity of the structural model for the direct effect. Second, the maximum likelihood method was conducted to estimate the parameters and fit of the constructed structural models to test Hypotheses 1, 2, and 3.

The analysis of the moderating model includes two parts. In the first part, the study sample was separated into two clusters according to the 1% dividing line of the elderly-to-social worker ratio, one comprising the high-ratio group and the other comprising the low-ratio group. Multi-group confirmatory factor analysis (MGCFAs) was performed to test the measurement invariance to ensure the measurement instrument operates consistently across the high-ratio and low-ratio groups [41]. MGCFAs started with testing the basic level of invariance of the configural model, which imposes no equality constraints across the groups. Then additional constraints were added by restricting factor loading and intercepts equally across the groups. In the second part, moderating effects were assessed by critical ratio of difference analysis to test Hypotheses 4, 5, and 6.

Since a single index for measuring goodness-of-fit has various limitations, this study adopts several fit indices for a more comprehensive evaluation of the statistical model. These indices include the ratio of chi-squared to degrees of freedom (χ²/*df*), the comparative fit index (CFI), the goodness-of-fit index (GFI), and the root mean squared error of approximation (RMSEA). For the χ²/*df* ratio, since the values for χ² and significance level vary greatly according to sample size [42], adequate model fit is indicated by a χ²/*df* ratio between 1 and 5 [43]. As for the other indices, values of CFI and GFI higher than 0.90 suggest a good model fit, while an RMSEA value lower than 0.08 suggests an acceptable model fit [44]. For tests of measurement invariance, the practical acceptable criterion of difference for CFI (ΔCFI) was used as an index. The value of ΔCFI smaller than or equal to -0.01 indicate strong invariance [45]. Multi-group analysis produced results for the moderation effect using a critical ratio of greater than 1.96 [46].

## Results

### Descriptive statistics and correlations

Table 2 presents the mean scores, standard deviations, and correlation coefficients among the primary variables. The results show that institutional caregiver interaction, family caregiver interaction, and perceived control are all positively correlated with meaning in life.

**Table 2:** Descriptive statistics and bivariate correlations for key variables

	M	SD	1	2	3	4
1. Institutional caregiver interaction	59.02	9.290	1			
2. Family caregiver interaction	4.05	1.215	0.300^**^	1		
3. Perceived control	23.28	4.687	0.426^**^	0.203^**^	1	
4. Meaning in life	18.62	4.569	0.534^**^	0.283^**^	0.441^**^	1

Note: *p < 0.05, **p < 0.01

### Results of structural equation modeling

#### Results of CFA

The CFA results for institutional caregiver interaction showed a good model fit (χ²/*df =* 2.516, *p* < 0.001, CFI = 0.995, GFI = 0.981, RMSEA = 0.058, standardized factor loading range = 0.823–0.895). Similarly, the CFA results for meaning in life also showed a satisfactory model fit (χ²/*df* = 0.797, *p* < 0.001, CFI = 1.000, GFI = 0.997, RMSEA = 0.000, factor loading range = 0.504–0.934). According to the cut-off scores for reliability [47], both variables may potentially represent latent constructs.

#### Results of the direct effects model

Table 3 presents the results of the structural model showing direct effects after controlling for gender, age, duration of stay, education level, marital status, and income level, which shows an adequate fit (χ²/*df* = 2.254, *p* < 0.001, CFI = 0.959, GFI = 0.914, RMSEA = 0.053). Both institutional caregiver interaction (*β* = 0.365, *p* < 0.001) and perceived control (*β* = 0.259, *p* < 0.001) have positive impact on meaning in life. However, no significant relationship was found between family caregiver interaction and meaning in life (*β* = 0.100, *p* > 0.05). These results indicate that high levels of institutional caregiver interaction and perceived control are correlated with a greater sense of meaning in life, whereas the family caregiver interaction is not related to sense of meaning in life.

Among the control variables, the education level of elderly residents is significantly related to their sense of meaning in life (*β* = 0.128, *p* < 0.001), revealing that residents with higher educational backgrounds tend to have a stronger sense of meaning in life. Moreover, age is significantly associated with meaning in life (*β* = 0.098, *p* < 0.05), indicating that older residents experience a greater sense of meaning in life compared to younger residents.

**Table 3:** Unstandardized and standardized path coefficients of the structural model

Influencing path	Estimate	SE	β	C.*R*.	*p*
1. Institutional caregiver interaction → Meaning in life	0.246	0.041	0.365	6.043	***
2. Family caregiver interaction → Meaning in life	0.148	0.087	0.100	1.694	0.090
3. Perceived control → Meaning in life	0.139	0.030	0.259	4.662	***

Note: Estimate, unstandardized path coefficient; *SE*, standard error; *β*, standardized path coefficient; C.R., critical ratio; *p*, significance level

***p < 0.001

#### Results of MGCFAs

As shown in Table 4, after fitted the sample into the high-ratio and low-ratio groups simultaneously, the configural invariance model (M1) showed a reasonable fit (χ²/*df* = 1.643, *p* < 0.001, CFI = 0.975, RMSEA = 0.038), and all factor loading were found to be significant (*p* < 0.05). In terms of the metric invariance model (M2), the results reveal a good model fit (χ²/*df* = 1.665, *p* < 0.001, CFI = 0.973, RMSEA = 0.038). The difference between the M2 and M1 was not practical significant with ΔCFI=-0.002<-0.01, indicating that the factor loading are equivalent between the two groups. Subsequently, the analysis results of scalar invariance model (M3) exhibit acceptable fit with the data (χ²/*df* = 1.782, *p* < 0.001, CFI = 0.966, RMSEA = 0.042), ΔCFI=-0.007<-0.01 suggesting that the intercepts were equivalent across the two groups. In sum, the above results presented support for measurement equivalent across high and low elderly-to-social worker ratio groups.

**Table 4:** Tests of measurement invariance across elderly-to-social worker ratio groups

high ratio vs. low ratio	χ²/df	RMSEA	CFI	Model comparison	ΔCFI
M1 (no constrains)	1.643	0.038	0.975	-	-
M2 (equal factor loadings)	1.665	0.038	0.973	2 vs. 1	-0.002
M3 (equal intercepts)	1.782	0.042	0.966	3 vs. 2	-0.007

#### Results of moderation analysis

**Fig. 1:**
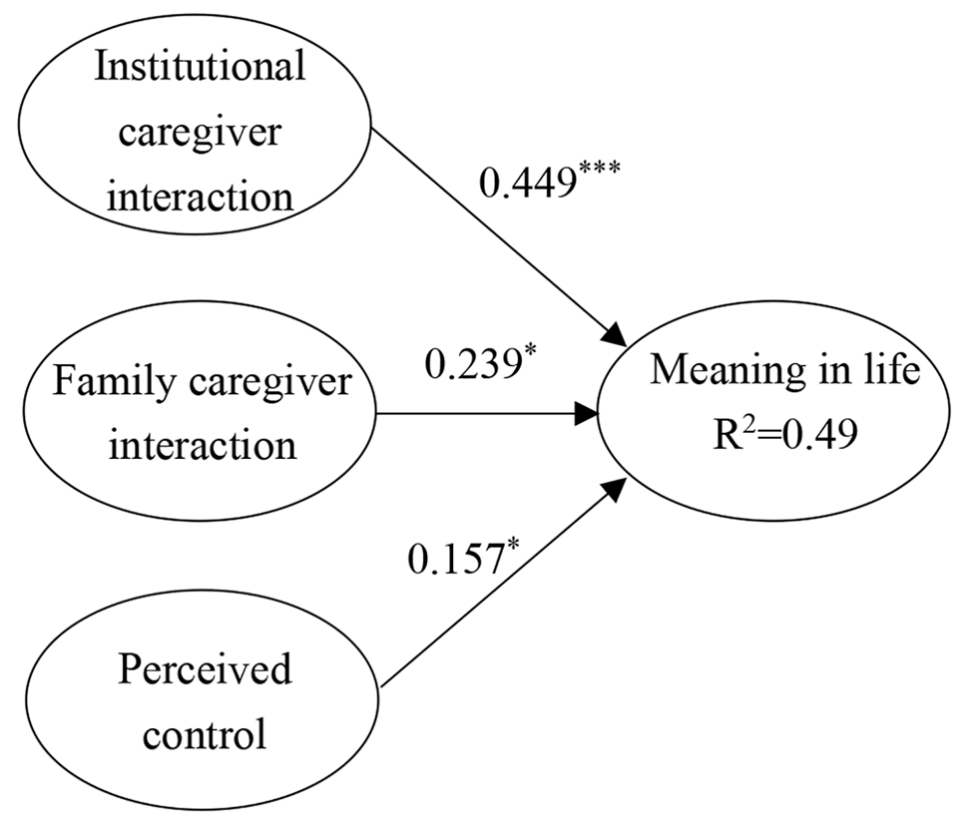
Effects for social worker high ratio group

**Fig. 2:**
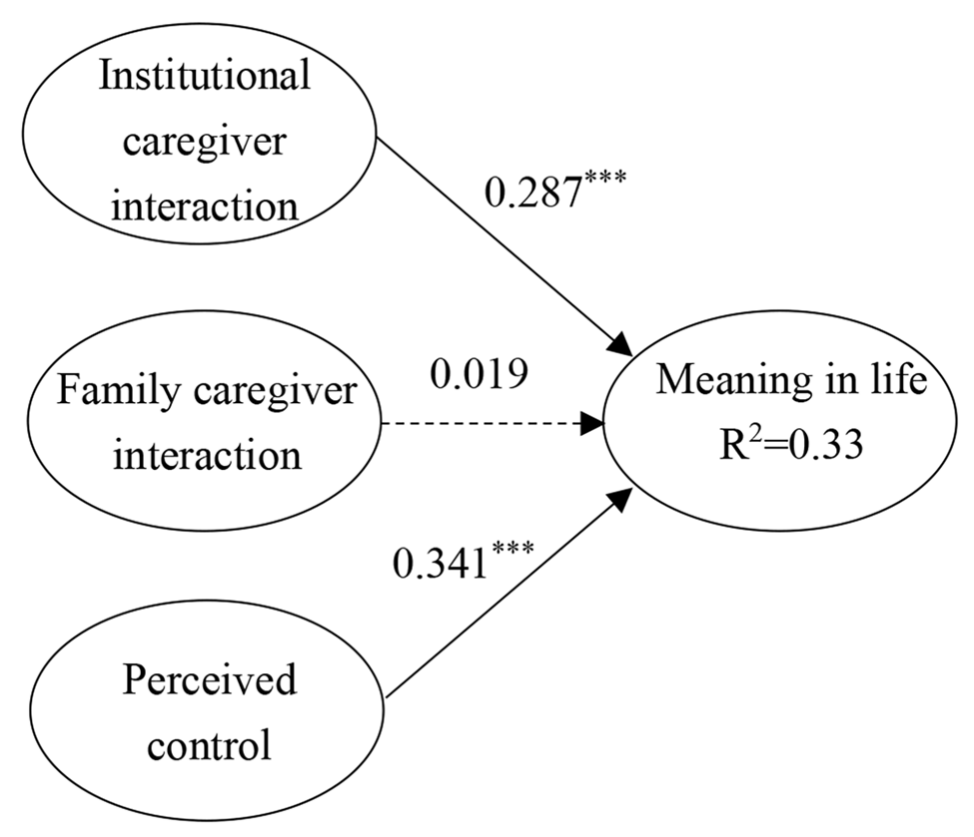
Effects for social worker low ratio group

Based on the direct effects results, the moderation analysis selected the education level and age of the elderly residents as control variables. The moderation model showed a good model fit (χ²/*df* = 1.584, *p* < 0.001, CFI = 0.971, GFI = 0.900, RMSEA = 0.036). For the high-ratio group (see Fig. 1), institutional caregiver interaction (*β* = 0.449, *p* < 0.001), family caregiver interaction (*β* = 0.239, *p* < 0.05), and perceived control (*β* = 0.157, *p* < 0.05) all promoted a sense of meaning in life. For the low-ratio group (see Fig. 2), institutional caregiver interaction (*β* = 0.287, *p* < 0.001) and perceived control (*β* = 0.341, *p* < 0.001) promoted a sense of meaning in life, whereas family caregiver interaction did not.

**Table 5:** Standardized path coefficients of the subsamples with higher and lower elderly-to-social worker ratio

Influencing path	Social worker high ratio group (*N* = 231)			Social worker low ratio group (*N* = 221)			C.*R*. differences
	SE	β	C.*R*.	SE	β	C.*R*.	
1. Institutional caregiver interaction → Meaning in life	0.063	0.468^***^	5.766	0.052	0.289^***^	3.317	-2.195
2. Family caregiver interaction → Meaning in life	0.256	0.244^*^	2.426	0.113	0.006	0.062	-2.319
3. Perceived control → Meaning in life	0.049	0.143	1.915	0.043	0.345^***^	3.723	1.006

*p < 0.05, ***p < 0.001

As shown in Table 5, results revealed that the elderly-to-social worker ratio moderated the relationship between institutional caregiver interaction and meaning in life, suggesting that a higher ratio could promote the effects of institutional caregiver interactions on meaning in life. Similarly, the ratio also moderated the relationship between family caregiver interaction and meaning in life, suggesting that a higher ratio could strengthen the relationship between family caregiver interactions and meaning in life.

## Discussion

This study aimed to fill a gap in the literature regarding the moderating role of the elderly-to-social worker ratio in the associations between institutional caregiver interactions, family caregiver interactions, perceived control, and perceptions of the meaning in life among elderly care residents. The findings demonstrate that the relationships that elderly residents have with institutional caregivers and their perceived control promote a sense of meaning in life. Moreover, the moderating effect of the elderly-to-social worker ratio at the organizational level affect the relationships between institutional caregiver interactions, family caregiver interactions, and meaning in life. As discussed below, the study findings partially support research hypotheses.

### Caring relationships and meaning in life

The present study found evidence that institutional caregiver interactions have a positive impact on meaning in life among the elderly, supporting H1. In the ethics of care theory, the key elements that determine the quality of professional caring interactions include respect, acceptance, and understanding attitudes during the service process [48]. Research indicates that daily interactions between care staff and elderly residents not only directly affect resident’s physiological health but also their mental health status, providing evidence for the importance of establishing professional caring relationships in institutional care. Such a professional connection does not only make the elderly residents more dependent; instead, it makes them feel supported and cared for during interactions, thus promoting the overall wellbeing of care recipients [49].

Conversely, the present study found that family caregiver interactions do not directly predict perceptions of meaning in life among elderly residents, contrary to the prediction made in H2 and inconsistent with existing literature on Asian community-dwelling elderly populations. The unexpected finding can be attributed to the development of meaning in life under the life course perspective. According to this theory, family is no longer an important life goal to be completed for an elderly person after moving into a nursing home, but rather one that has already been achieved, which provides satisfaction and achievement [50]. Moreover, evidence shows that family has no significant influence on perceptions of the existence of meaning in life among elderly nursing home residents in western Europe, both individuals with normal cognition and those with Alzheimer’s disease [51].

Although Chinese culture emphasize interdependent self and familial connectedness, older adults often feel lonely due to the lack of family involvement after having been admitted to the care home [52]. On the one hand, the elderly who can receive support from a harmonious family are less likely to live in institutional care [53]; on the other hand, the accessibility of elderly homes such as location, traffic or physical environment might act as barriers to family caregiver interaction with the elderly. Elderly residents tend to believe that their responsibility for family has been completed before moving into the elderly home, and no longer feel the need to rely on their family members. Therefore, the family caregiver interaction has no significant effect on the meaning in life of the elderly.

Regarding perceived control, the present study found evidence that it has a positive impact on meaning in life among the elderly, supporting H3. For elderly people with long-term care needs, a sense of control in life is a key dimension that the ethics of care theory addresses [54]. In the later stages of life, the elderly with a higher perception of control are at a lower risk of mortality [55]. Elderly with stronger perceptions of control are more positive when facing physiological changes. They are also more likely to maintain their preferred living habits after moving into residential care, which may improve their life satisfaction. When elderly people have a greater sense of perceived control, they are more capable of using relevant resources to overcome obstacles in a challenging environment through their own efforts, which positively affects their sense of meaning in life [56].

Finally, the present study found evidence that the elderly-to-social worker ratio moderates the associations of institutional and family caregiver interactions with meaning in life, supporting H4 and H5, respectively. While several researchers have found a positive correlation between the number of nursing staff and service outcomes in care institutions [57, 58], few studies have paid attention to the ratio of social work staff at these institutions. The service team who directly provide care to the elderly are often composed of multi-professionals. And the quality for multi-professional’s cooperation requires as well to consider the number of social workers, as they facilitate communication, autonomy for professionals, safe team culture, and suitable care planning [59]. Insufficient elderly-to-social worker ratio will lead to heavy workload, which was one of the most common barriers for developing a closer connection of staff to residents [31]. Because residents and families are struggling with difficulty to find meaning in life in care facilities, they need adequate social workers to build the supporting system for their daily lives. Thus, the suitable elderly-to-social worker ratio not only affects the financial management, but also improves the service efficiency.

The moderating analysis results indicate that the elderly-to-social worker ratio does not have a significant moderating effect on perceived control and meaning in life. This finding may highlight the shortcomings of social workers in fostering a humanistic service atmosphere in long-term care facilities. Social workers might prioritize perceived control during service delivery at the interpersonal level, inadvertently overlooking the deprivation of elderly individuals’ sense of control within institutional regulations at the structural level. Due to the necessity of complying with institutional regulations in the service delivery process, there is very limited space for staff to offer the elderly a sense of autonomy. To foster humanistic care, social workers need to prioritize the rights of the elderly both on an interpersonal and structural level.

In China, only very few institutions employ full-time social workers, which reveals that the government do not attach sufficient importance to people’s mental health in long-term care. However, the social worker could improve the mental health status of elderly residents, respond to families’ concerns, and contribute to the outcome of cross-disciplinary cooperation [60, 61]. Although social workers in institutional care have an important role in supporting the care residents, they are often underused and overlooked over the course of facilitating care delivery from family and staff. An adequate elderly-to-social worker ratio not only increases the effectiveness of interventions on the psychological needs of elderly residents, but also promotes the positive impact of family caregiver interactions on their mental health statuses. Since social workers play a critical role in promoting the relationship between caregiver interactions and meaning in life, the elderly-to-social worker ratio is an important factor promoting the living status of elderly residents.

### Implications for policy and practice

The concept of meaning of life provides a direction for addressing elderly resident’s mental health in institutional care. Since the landscape in institutional care has shifted dramatically, resident needs will continue to grow in complexity due to changes in life expectancy and more widespread recognition of mental illness [62]. Governments have the responsibility to guide healthcare institutions, as well as allocating more resources for mental health and social services. Governments should also encourage these institutions to employ an adequate number of professionally certified social workers through establishing a reasonable staff ratio standard in welfare policy, in addition to attracting more qualified social workers to work in institutional care by improving the payment for social workers, which can improve the efficiency of institutional care services.

Operational guidelines for long-term care services should not just be limited to standards for service procedures or task completion, but also pay attention to staff attitudes and skills during their interactions with elderly residents in care facilities. When establishing service standards and management regulations, institution managers should keep an open mind toward the actual needs of elderly residents, as well as encourage positive cooperation between social work services and other care services. The findings of this research encourage care facilities to fully leverage the strengths of social workers in coordinating social activities, facilitating caring relationships, and creating a humanistic environment. Social workers should also take the initiative to establish a support system between elderly residents and institutional andfamily caregivers to improve their sense of meaning in life.

### Limitations

Although this research has made some theoretical and practical contributions, it also has the following limitations that must be addressed. First, the sample for this study was selected from the population of elderly care institutions with social work services in urban areas; concerns about generalizability arise when the research conclusions are applied to institutions without social workers or institutions in rural areas. Moreover, the cross-sectional data collected through the measurement instruments can only verify correlations between variables, and cannot identify causal relationships between independent and dependent variables. Finally, since the development of institutional care service mechanisms for spiritual wellbeing services is an ongoing process, further research may consider incorporating qualitative analysis to enhance understandings of meaning in life.

## Data Availability

The datasets generated and analyzed during the current study are not publicly available to protect the participates’ privacy, but are available from the corresponding author upon reasonable request.
